# Principles, Scope, and Limitations of the Methodological Triangulation*

**DOI:** 10.17533/udea.iee.v40n2e03

**Published:** 2022-09-15

**Authors:** María Mercedes Arias Valencia

**Affiliations:** 1 Nurse, Ph.D. Professor at Facultad de Enfermería, Universidad de Antioquia. Medellín (Colombia). Email: mercedes.arias@udea.edu.co Universidad de Antioquia Facultad de Enfermería Universidad de Antioquia Medellín Colombia mercedes.arias@udea.edu.co

**Keywords:** qualitative research, methodology, observation, investigación cualitativa, metodología, observación., pesquisa qualitativa, metodologia, observação.

## Abstract

This article sought to collect basic and relevant information about methodological triangulation and make a first approach to the principles underlying its use, potentiality and scope, advances and limitations, and some alternative proposals to surpass them. In that sense, it is an attempt to operationalize concepts and present the procedures to conduct it rigorously. In the first place, conceptual aspects and types of triangulation are presented, and in the second place, the principles, uses and difficulties. But, beyond what must be done, an approach is made to how to do it. The assumption underlying through the article is the complementarity among methods. It is emphasized in the principle through which the nature of objects must guide the selection of the methods and of the most effective techniques to approach and account for phenomena that are socially pertinent of being studied.

## Resumen

El presente artículo pretende levantar información básica y relevante sobre la triangulación metodológica y hacer una primera aproximación a los principios que subyacen en su uso, su potencialidad y alcance, sus avances y limitaciones, y algunas propuestas alternativas para superarlas. En ese sentido, es un intento de operacionalizar los conceptos y presentar los procedimientos para llevarla a cabo en forma rigurosa. En primer lugar, se presentan los aspectos conceptuales y los tipos de triangulación, y en segundo lugar los principios, los usos y las dificultades. Pero, más allá del qué hacer, se hace una aproximación al cómo hacerlo. El supuesto que subyace a través del artículo es la complementariedad entre los métodos. Se enfatiza en el principio mediante el cual, la naturaleza de los objetos debe guiar la escogencia de los métodos y de las técnicas más eficaces para aproximarse y dar cuenta de los fenómenos que son pertinentes socialmente, de ser estudiados.

## Resumo

Este artigo tem como objetivo coletar informações básicas e relevantes sobre triangulação metodológica e fazer uma primeira aproximação aos princípios que fundamentam sua utilização, seu potencial e alcance, sua avanços e limitações, e algumas propostas alternativas para superá-los. Nesse sentido, é uma tentativa de operacionalizar os conceitos e apresentar os procedimentos para realizá-lo com rigor. Em primeiro lugar, são apresentados os aspectos conceituais e os tipos de triangulação e, em segundo lugar, os princípios, usos e dificuldades. Mas, além do que fazer, é feita uma abordagem de como fazer. A hipótese subjacente ao longo do artigo é a complementaridade entre os métodos. A ênfase é colocada no princípio pelo qual a natureza dos objetos deve orientar a escolha dos métodos e técnicas mais eficazes para abordar e dar conta dos fenômenos socialmente relevantes, se estudados.

## Introduction

According to Boudon,^1^ for authors, like Dilthey, Rickert, Jaspers, and Max Weber, research in social sciences follows the path of understanding and the natural sciences through explanation, although for some, especially for Weber, both procedures, although distinct, are not exclusive. The same author found false opposition between the methods of the sciences, given our condition of social beings and the specificities of the human, through the diversity of objects and limitations of the methods, to account for complex phenomena of the social reality. For this author, it is naive to evaluate the methods of the social sciences with the unified parameters of the natural sciences, given that it would not be imaginable, for example, that History could be similar to Physics.

*Quantitative research* is supported on a set of established logical principles and should not be imposed from the outside for the researcher. *Qualitative research* also obeys an implicit but less unifiable logic.^1^ The nature of the object and effectiveness of the methods will guide the researcher’s reflection to approach and account for phenomena that are pertinent, socially, of being studied. It must be highlighted that the methods are not the truth, they only constitute tools, procedures, instruments and modes of putting together the theory to investigate a problem and that when used facilitate its understanding; in that sense, the methodological triangulation will be treated as research procedure.

The term triangulation comes from navigation, where, from various angles, an object is situated; in this case, a ship. Thus, triangulation constructs several appendages, namely theoretical or methodological perspectives, several views or several readings, diverse points of view to address the same research problem. As explained by Morse, the discussion among authors has dealt on the appropriations, advantages, and disadvantages of methodological triangulation.^2^ The issue that has gained greater interest is the combination of qualitative and quantitative methods within the same project. Some authors have published examples of how this is carried out within a specific project, identifying the issues involved in said strategies; others have identified unsolved issues or highlight the guidelines they consider successful and the less developed in the use of methodological triangulation.

This article sought to collect basic and relevant information about methodological triangulation and make a first approach over the principles underlying its use, potentiality and scope, its progress and limitations, as well as solution alternatives.

## From triangulation of indicators and variables to theoretical and methodological triangulation: conceptual aspects

What is methodological triangulation? Triangulation is a term originally used in navigation circles by taking multiple reference points to locate an unknown position. Campbell and Fiske are credited in the literature as the first to apply triangulation in research in 1959.^3^ It is assumed conventionally that triangulation is the use of multiple methods to study the same object. This is the generic definition, but it is only one form of the strategy. It is convenient to conceive triangulation including varieties of data, researchers and theories, as well as methodologies.^4^

Kimchi *et al*.,^5^ assume the definition by Denzin in 1970 on triangulation in research: it is *the combination of two or more theories, sources of data, research methods, in the study of a singular phenomenon.* Close scrutiny reveals that the combination can be interpreted in several manners; for such, the authors start from the classification by Denzin and provide explanations about the most adequate way of performing it.

For Cowman,^3^ triangulation is defined as the combination of multiple methods in studying the same object or event to better address the phenomenon researched. In turn, Morse^2^ defines *methodological triangulation* as the use of at least two methods, usually qualitative and quantitative, to guide the same research problem. When a singular research method is inadequate, triangulation can be used for a more comprehensive approach to solve the research problem.

## Multiple triangulation strategies

Denzin^4^ describes four basic types of triangulation: 1) data triangulation with three subtypes of time, space and person; the person analysis, in turn, has three levels: aggregate, interactive and collective; 2) researcher triangulation that consists in using multiple observers, more than single observers of the same object; 3) theoretical triangulation that consists in using multiple perspectives, *more* than single perspectives in relation with the same *set* of objects, and 4) methodological triangulation that can imply triangulation within methods and triangulations among methods.

### Data triangulation^4^

Denzin^4^ illustrates this type of triangulation. For the author, observers can triangulate with data sources and researchers make explicit the search for the different sources. For example, analysts can employ, in efficient manner, the same methods for a maximum theoretical advantage. Thus, for example, in studying the social meaning of death in a modern hospital it may be possible to use a standard method (like participant observation, which, in strict manner would be technical) and deliberately follow this method in as many different areas as possible.

Researchers can observe different groups within the hospital and take the family members of the dead people. Death rituals can also be examined with the same process. Other examples are deaths on the road, deaths at home, deaths at work and even deaths at play. Each represents a different area of significance with which the same generic event (death) occurs. Basically, this could be used in a comparison of dissimilar groups as a sampling strategy, but more properly reflects a triangulation strategy. Selecting different collocations systematically, researchers can discover that its concepts (like assignment of reality units) share common issues. Similarly, the constituent unit of those concepts can be discovered in its contextual situation.

Furthermore, all sociological observations report activities of people situated socially -although they are in groups or organizations or distributed in groups in a social *area*-. Focusing time and space as observation units recognizes their relationship with the observations of people. Observers can make a sampling of activities according to time of day, week, month or year. Likewise, they can do it with space and treat it as an analysis unit (for example, ecological analysis), or as a component of external validity. The most-common analysis unit, the social organization of people can be sampled over time and space. Those three units -time, space and person- are interrelated. Studying one demands studying the others.

*Levels of person analysis.* Three levels of person analysis can be treated:^4^

1. Aggregate analysis. It is the first level; selecting individuals for the study, not groups, or relationships, or organizations. This level of analysis is called aggregate because it does not establish social relationships among that observed. Random samples of house workers, school students, and laborers are examples of aggregate analysis of persons.
2. Interactive analysis. It is the second level and is related directly with the symbolic interaction. Regarding the term interactive, a unit exists among people interacting in the laboratory or in the natural field. For example, small groups, families or aviators. Sociologists commonly associate it with participant observation; experiments in small groups and non-obstructive measurements represent this form of analysis. The unit is the interaction more than person or group; for example, face-to-face studies by Goffman, who investigated in insurers, nurses and hospital social structure, only how they interact in the generation of series of interactive episodes.
3. Collective analysis. The third level, more commonly associated with the structural-functional analysis, is the collectivity. Here, the observational unit is an organization, group, community or, even, an entire society. People and their interactions are treated only according with how they reflect pressures and demands of the total collectivity.

The three levels of analysis may be illustrated by returning to the example of death in hos pital. Research guided in aggregate manner can sample simply the attitudes of the hospital staff during the process. An interactional study can examine how those attitudes are generated by the encounters of the personnel. Lastly, the researcher aimed towards the collectivity can examine how the hospital’s structural units (for example, its organizational charter, job positions) dictate certain attitudes and practices by its members.

In synthesis, any research can combine the three levels and types of data; in effect, those studies commonly recall as classical events these combinations: time, space and person are alternatively analyzed in the aggregate, interactive, and collective levels.

### Researcher triangulation^4^

Researcher triangulation means multiple observers are used, rather than a single one. More researchers, in effect, conduct multiple observations, although not all play equally prominent roles in the process. Delegation at work can be established by placing well-prepared individuals in crucial positions. When using multiple observers, the most skilled should be placed near to the data. Upon triangulating observers, potential bias coming from single person is removed and considerable reliability is ensured in the observations.

There are various field workers subjected to the same data. If a colleague reports the same class of observation as another, without prior consultation, trust is increased. If later, listening to the report of an observation, a colleague contributes the same, unquestionably duplicates it; that indicates that our observation techniques have some degree of reliability.

Multiple observers may not agree on what they are observing, given that each observer has unique interactional experiences with the phenomenon observed.^4^ Researcher triangulation is considered present when two or more trained researchers with divergent antecedents explore the same phenomenon. It is considered to take place when; 1) each researcher has a prominent role in the study, 2) the experience of each researcher is different, and 3) the disciplinary bias of each researcher is evident in the study. This definition, as the previous classifications, was elaborated and extended by Denzin in 1989, who stated that researcher triangulation occurs when two or more skilled researchers examine the data. The concern that stands out from researcher triangulation is that different disciplinary biases are compared or neutralized through the study. Overall, this is not discernible in a research publication. Researcher triangulation is difficult to distinguish, unless the authors describe explicitly how they achieved it.

### Theoretical triangulation^4^

Denzin defined theoretical triangulation as an evaluation of the usefulness and being able to test rival theories or hypotheses. This definition includes tests through research, rival theories, rival hypotheses or alternative explanations of the same phenomenon. Denzin placed as example the studies by Campbell of women’s responses toward abuse, which provide an example of theoretical triangulation. Two competitive models were tested in the same sample of women. Both were used previously to explain the women’s responses. The goal was to pit them against each other in a singular study to determine which one provides the best explanatory model of the phenomenon of abuse. The data collection approached was used to measure specific concepts and variables from each model. The report published placed the objective *a priori,* to the test of two opposing rival theories; this component is necessary to operationalize the theoretical triangulation.

Theoretical triangulation is an element few researchers manage and end up reaching. Overall, a small group of hypotheses guides the study and the data obtained emerge not only in those dimensions, rather they may appear with value, in empirical approach materials with multiple perspectives and interpretations in mind. Data could refute the central hypothesis and various theoretical points of view can take place to determine its power and usefulness. Each strategy can allow the contribution of criticism and controversy from several theoretical perspectives. Confronting theories in the same body of data means the presence of efficient criticism, more in line with the scientific method. This last issue can be qualified by understanding, for example, that sociologists never have the same body of data; this means that a body of data of empirical materials is always socially constructed and subject to multiple interpretations.

### Methodological triangulation

Triangulation of methods using two or more research methods can be made in the design or in the data collection. Two types exist, triangulation within methods and among methods.^4^

Triangulation within methods is the combination of two or more data collections to approach the study of the same object; using two or more quantitative measurements of the same phenomenon in a study is an example. Including two or more qualitative approaches, like the observation and open interview to assess the same phenomenon, is also considered triangulation within methods. Observational data and interview data are coded and analyzed separately, and then compared, as a way of validating the findings.

This form is used more frequently when the observational units are seen as multidimensional. Researchers take a method (from safety) and employ multiple strategies to examine the data. A safe questionnaire can be constructed with different measurement scales for the same empirical unit. For example, in the famous case of the alienation scales, several recent investigations have used five different indices. The obvious difficulty is that only one method is employed. Observers are mistaken if they believe that five different variations on the same method generate five triangulation varieties.

Moreover, each class of data generated -interviews, questionnaires, observation and physical evidence- is potentially biased and its specificity may be threatened. Ideally, data should converge, *i.e.*, they should not contradict, although conserving their multiple variations. 

Triangulation among methods is a more sophisticated way of combining triangulation of dissimilar methods to illuminate the same class of phenomena; it is called among methods or triangulation through methods. The rationale in this strategy is that the weaknesses of a method constitute the strengths of another; and with a combination of methods, observers reach the best of each, overcome its weakness. Triangulation among methods can take several forms, but its basic characteristic can be the combination of two or more research strategies in studying the same empirical unit or several.

With seven research methods on research design -that in a stricter sense, would be techniques, a variety of combinations can be constructed.^1^,^2^ Completely triangulated research can combine them all. Besides, if the basic strategy was participant observation, researchers can employ safe interviews with field experiments, non-obtrusive methods, filming, and life stories. Most sociological research can be seen to emphasize a dominant method, with combinations of other additional dimensions.

Kimchi *et al*., state in their article Denzin’s classification and add explanations about the most adequate way of conducting the triangulation.^5^ In their opinion, the specificity and the step-by-step procedures to implement the triangulation should be addressed. The purpose of their work was to present operational definitions for the types of triangulation described by Denzin in an effort to clarify the triangulation and attract researchers. Based on the theoretical definitions by Denzin, these show a group of operational definitions of the types of triangulation. The definitions seek to clarify, specify, and provide indicators that research readers can use if they deem there has been triangulation. Operational definitions were made by Kimchi during a review of all the data on which 319 articles were based from six nursing research journals published during 1986 and 1987. The six journals were: *Advances in Nursing Science, Image, Inter national Journal of Nursing Studies, Nursing research, Research* in *Nursing and Health, Wes tern Journal of Nursing Research.* The following presents some operational definitions.

- Data triangulation.^5^ Considered as the use of multiple data sources to obtain diverse visions about a topic for the purpose of validation. Temporal triangulation represents data collection of the same phenomenon during different points over time, as already exposed; in these studies, time is relevant. Longitudinal studies are not considered temporal triangulation because the aim of a longitudinal study is to document changes over time and the purpose of temporal triangulation is to validate the congruence of the same phenomenon through different points over time.

- Spatial triangulation.^5^ It is data collection of the same phenomenon in different sites. Space must be the central variable. Studies in which data are collected in multiple sites, but do not cross, are not considered spatial triangulation. In spatial triangulation, data are collected in two or more scenarios and tests of consistency are analyzed by crossing the sites.

-Person triangulation.^5^ It is data collection from, at least, two of the three levels of person: individuals, couples, families, groups or collectives (communities, organizations or societies). Researchers can collect data from individuals, couples and groups, or each of the three types. Data collection from a source is used to validate data from the other sources or a single one. Kimchi, Polivka and Stevenson set as example the work by Hutchinson who, in 1987, studied the process of dependency on recovery ward nurses on two levels. Data were collected weekly from meetings of groups of recovery nurses over one year (group level) and in selection interviews (individual level). The phenomenon of interest was the recovery process. Each data level was used to validate the findings of the other.

- Multiple triangulation.^5^ This occurs when using more than one type of triangulation in analyzing the same event, contributing more comprehensive and satisfactory sense of the phenomenon^4^; as mentioned, it is the combination of two or more types of triangulation in a study. Using triangulation within methods and researcher triangulation in a study or using triangulation within methods and among methods in a study are two examples of multiple triangulation. Kimchi *et al*., give as an example the study by Wallson *et al*., which combined researcher triangulation and triangulation within methods. The group represents a multidisciplinary mix of researchers and study goals reflected on distinct values from different disciplines. Triangulation within methods was evidenced by the use of three measures of stress, each used to validate the others, a psychological measure and two written tests.

Triangulation in the analysis, a more recent type of development, is the use of two or more approaches in the analysis of the same data group for validation purposes. It is conducted by comparing data analysis results, using different statistical tests or different techniques of qualitative analysis to evaluate similarly the results available. It serves to identify similar patterns and, thus, verify the findings. Use of divergent methods of data analysis for cross-validation purposes constitutes another triangulation potential. For Denzin,^4^ “*the greatest goal of triangulation is to control the personal bias of researchers and cover the intrinsic deficiencies of a single researcher or a unique theory, or the same method of study and, thus, increase the validity of the results*”.

- Combination of results: Morse^2^ agrees with Mitchell in that the problem of the weight of the results of each component is solved if the findings are interpreted within the context of present knowledge. Each component should fit as a piece in a puzzle. The essential is the process of informed thought, judgment, wisdom, creativity, and reflection, and includes the privilege of modifying the theory, this is the exciting part of each research project and when there is triangulation of different methods, this is particularly exciting. If contradictory results occur from the triangulation of qualitative and quantitative methods, then a group of findings is invali d or the total result of the study is inadequate, incomplete or imprecise or both. If the study was guided deductively, the theoretical map may be incorrect.

## Implementing the methodological triangulation 

The methodological triangulation can be classified as simultaneous or sequential.^2^ The first, when using qualitative and quantitative methods at the same time. In that case, the interaction between both data groups during the collection is limited, but the findings complement each other at the end of the study. Sequential triangulation is used if the results of a method are essential to plan another method. The qualitative method is completed before implementing the quantitative method or vice versa.

Thus, according to Morse,^2^ in the methodological triangulation, the key issue is if the theory, which guides the research, is developed inductively or is used deductively, as in the quantitative inquiry. From this differentiation, various types of methodological triangulation result. If the research is directed by an inductive process and the theory is developed qualitatively and is complemented through quantitative methods, the *QUAL + quan* notation is used to indicate simultaneous triangulation. If the project is deductive, directed by a conceptual map *a priori,* the quantitative methods take precedence and can be complemented with qualitative methods. In that case, the *QUAN + qual* notation is used*.* The sequential triangulation is indicated by *QUAL*-› *quan* with an inductive project, that is, when the theoretical direction is inductive and uses a qualitative foundation. Using the *QUAN*-› *qual* notation indicates a deductive approach; that is, when we follow the complete quantitative steps and the qualitative method is used to examine or explore unexpected encounters. 

### Principles

The purpose of the article by Morse^2^ was to explore the principles underlying the use of methodological triangulation when combining qualitative and quantitative methods. Those principles are related with the consistency among the research purpose, research problem, method used, sample selection, and interpretation of the results. The author coincides with Mitchell who highlights five areas of concern: 1) difficulty to combine text and numerical data; 2) interpretation of divergent results obtained from using qualitative and quantitative methods; 3) success or not in delineating and mixing the concepts; 4) weight of the information from different data sources, and 5) difficulty of guessing the contribution of each method when the results are similar. 

The first step in the quantitative-qualitative triangulation is to determine the nature of the research problem, if it is “natural” or “social”, which aims towards a primarily quantitative or qualitative approach. Characteristics of a qualitative research problem: 1) the concept under study is immature due to weak success and conspicuous theory and prior research; 2) a notion that the available theory may be inappropriate, incorrect or biased; 3) a need exists to explore and describe the phenomenon and develop theory, or 4) the nature of the phenomenon is not appropriate for quantitative measurements.

If a research problem is quantitative, the characteristics described are not applicable. Researchers can locate substantial and relevant literature about the topic, create a conceptual map, and identify hypothesis to test. In this case, the research design is comparative or correlational, experimental or quasi-experimental. 

The qualitative and quantitative aspects of a research project cannot be weighed equally: besides, a project must be guided theoretically by qualitative methods incorporating a complementary quantitative component, or guided theoretically by a quantitative method incorporating a complementary qualitative component. The important point is that each method must be complete in itself, that is, all the methods used must appropriate rigor criteria. If qualitative interviews are conducted, this must be done as if this method were alone. The interviews must continue while saturation is reached, and the content analysis has to be carried out inductively, more than forcing the data within a category preconceived for the study. 

Further, triangulation may be used with different objectives, among them, the following:

Triangulation is linked by many authors with rigor and quality; in that sense, one of the expectations is to increase research rigor,^6^ thus, Flick^7^ highlights triangulation as “a way to promote quality in research”.
Triangulation as verification: for Patton,^8^ studies using multiple methods that analyze different types of data “provide cross validation”. A les common use of triangulation is to ensure the validity of the instruments. However, this approach should be cautious, testing an instrument before its implementation or establishing its validity during the pilot test.
Triangulation as completeness: for Patton^8^ “(…) qualitative and quantitative data can be combined fruitfully when these elucidate complementary aspects of the same phenomenon”.
Interdisciplinarity: Flick^9^ proposes the possibility of conducting a “systematic triangulation of perspectives”, which may imply “researcher triangulation as collaborative strategy”; this opens the possibility addressing at least the multi- or interdisciplinarity; as proposed by Janesick:^10^ I would wish to add a fifth type: “interdisciplinary triangulation”.

In synthesis, following Molina,^11^ triangulation can “(…) expand the research process to contribute to deeper and broader comprehension of the phenomenon, given that it adds “(…) rigor, amplitude, complexity, richness, and depth to any research”.

Mixed methods in research -perspective under development and emerging since the 1990s- emphasize on integrating different data sets, as highlighted by Creswell.^12^ The author starts from the labels and notations exposed by Morse who was the precursor of said nomenclature and Creswell proposes it to differentiate design categories or typologies possible to apply in said methods.^12^ Said combined methods “have extended rapidly through social and behavioral sciences”, as stated by Timans, Wouters, and Heilbron^13^ and “have developed linked to the triangulation concept”.^12^ Some authors denominate the singularly as mixed method.

## The complementarity of methods

Defining qualitative research as development of theories and generation of hypothesis, and quantitative research as modification of theories and tests of hypothesis, Field and Morse have identified the complementarity of both approaches.

For Morse,^2^ the biggest threat to validity is the use of inadequate or inappropriate samples. Perhaps due to reasons of convenience, researchers have sought to use the same subjects for both methods, qualitative and quantitative, although it is clearly inappropriate to exchange those samples. For example, quantitative research is based on large representative samples of the population randomly selected; adjustment of the sample is determined statistically, as well as its representativity of the whole population. In qualitative research, appropriation is in relation to how well the sample can represent the phenomenon of interest (for example, how much have the participants experienced the phenomenon and can articulate their experiences); the sample will be adequate when data saturation is enriched. Still, in light of the overall purpose of research, no reason exists (different from convenience) to use the same subjects for both samples. 

Clearly, when incorporating quantitative methods within a qualitative study, the qualitative sample may be inadequate for quantitative purposes. Lack of representativity of the qualitative sample selected in purpose is inappropriate and threatens the validity. Selection of the sample through the qualitative and quantitative components of a sequential (*QUAL -› quan*) or simultaneous (*QUAL + quan*) triangulation must be independent. Because the quantitative sample is inadequate and inappropriate for quantitative purposes, researchers must design a quantitative sample for the population. However, when the quantitative method is used to add more information about the qualitative sample (*QUAL + quan*), exceptions can be made if the norms so permit, or if a comparison is available of a normal group, to interpret the results. For example, if dealing with the anxiety of the relatives in the waiting room, the anxiety scales can be interpreted with the norms available for anxiety scales.

A subsample may be used from a large quantitative sample for the qualitative component of the *QUAN* + *qual or QUAL -› quan* triangulation*,* but those subjects included or the incidental observations in the qualitative part must be selected according with the criterion of good participants than through random selection. Thereby, the subjects selected for the quantitative sample must have greater experience and articulation, and the observations selected must consider the best examples of the situation.

Methodological triangulation is not a term applied to ethnography when the research method includes the use of semi-structured interviews, some levels of participant observation, use of recordings, and administration of questionnaires. It is the combination of said techniques that constitutes the ethnography and what makes ethnography, ethnography. It is not the case of blending or integrating guides from both texts, qualitative and quantitative, rather, it is using appropriate strategies to maintain the validity of each method. The *QUAN* + *qual* triangulation is not only the addition of linguistic and narrative data in an experimental design; at least, the interview data must be collected and analyzed according with the assumptions and principles of the qualitative method. Similarly, incorporating one or two open questions within the quantitative survey does not make study qualitative.

Additionally, using quantitative data in a qualitative study (like frequency data to improve the description), does not constitute a quantitative study. Methodological triangulation is not a technique to use due to rapidity and convenience in the research. Well done, it will likely lengthen the duration of the project, but the gains reached in the long term are immensurable.

Methodological triangulation is not a concurrent validation technique. Although the same strategies may be used, these are implemented in a study for different motives. The purpose of the concurrent validation is to find if the results of measuring the same concept through both methods are equivalent. The purpose of simultaneous triangulation is to obtain different but complementary data on the same topic, more than replicating the results.

According to Knafl, methodological triangulation is not merely to maximize the strength and minimize the weakness of each method. If a careful approach is not made, the end result may be to broaden the weakness of each method and invalidate completely the research project. It is more a method to obtain complementary findings and contribute to the theory and development of knowledge.

Some of the controversies of methodological triangulation have emphasized on the issue of qualitative research against quantitative. This controversy advocates for the combination of methods inasmuch as it is consistent with theoretical research. Some researchers forget that research methodologies are only tools, instruments that when used facilitate understanding. Researchers should be versatile and have a repertoire of methods available. To broaden the foregoing, a summary is presented of the discussion by Cowman about the paradigms and the author’s proposal regarding triangulation.^3^

Quantitative approach was the dominant paradigm from 1950 until 1990; the research approach - in turn - has been increasingly localized on the qualitative paradigm. Within the literature there is general support to separate both paradigms. However, accepting the inherent differences between the two, researchers are concerned that no isolated method can provide understanding of human beings and of their complex needs. Triangulation, as research strategy, represents the integration of two research approaches. The literature that explores its merits in research is incomplete, however, it is reported that triangulation, by reconciling the paradigmatic assumptions of quantitative and qualitative methods, provides richness and productive data. Triangulation offers a bipolar alternative and approaches the quantitative and qualitative. The qualitative-quantitative debate is still in development. It should be noted that each research perspective has several inherent differences. The quantitative approach has been associated exclusively with the dominant empirical-analytical paradigm and sees the causes of human behavior through observations that seek to be objective and collects quantifiable data. More often, research methods are associated with experimental research designs, which examine the causal relations among variables, controlled or removed from their natural scenario and observations are quantified and analyzed through statistically determined probabilities.

Quantitative research holds the methodological assumption that the social world looks at itself through objective forms of measurement. Conversely, Leininger 1985 suggests that people are not reducible to measurable objects and that they do not exist independently of their historical, social, and cultural context. The qualitative paradigm emerges from a tradition in sociology and anthropology, techniques to obtain qualitative data permit observing the world from the perspective of the subject, not the researcher. The qualitative paradigm is concerned for the value of the meaning and for the social world from which this meaning derives; through a variety of theoretical perspectives and research traditions that include phenomenology and ethnography, natural and family data are valued and serve to gain understanding of people. Differences between quantitative and qualitative approaches can be seen, even at the most basic level. The qualitative approach develops theory inductively from the data; in quantitative research, it is done deductively and its methods are encouraged primarily as a theory subjected to statistical tests, that is, falsifiable in Popperian terms.

Knowing the natural difficulties of research quantitative and qualitative methods and having identified the need to integrate the research approaches, the triangulation strategy is proposed. Cowman^3^ accepts four principles underscored by Mitchell,^14^ which, applied carefully, point to maximizing the validity of a particular research, incorporating the methodological triangulation: 1) the research question must be clearly focused, 2) the strengths and weaknesses of each method chosen must complement the other, 3) methods must be selected according with their relevance for the nature of the phenomenon under study, and 4) a continuous evaluation must be performed of the method selected during the course of the research to monitor if the three previous principles are being followed. These consistency elements also apply in mixed methods.

Cowman^3^ also warns of possible difficulties of triangulation: in first instance, a researcher, accepting the advantages of triangulation, can lose sight of differences between the methods chosen. Danger exists in collecting large volumes of data, which - subsequently - it will not be possible to analyze or are dealt with superficially. Fielding and Fielding emphasized on the danger of taking multiple methods without using simultaneously the bias control procedure.

Moreover, triangulation provides strengths, like animation, creativity, flexibility, and depth in data collection and analysis; as indicated by Cohen and Manion, methodologists often push methods as pets because those are the only methods with which they are familiar or because they believe that their method is superior to all the rest. Reichardt and Cook suggest that it is time to stop constructing walls between methods and start building bridges.

Given that the methods need independence within a single project, the real issue in triangulation can go beyond incompatibility between different assumptions of two paradigms, as argued by several researchers. It also assumes the possible incompatibility of contrasting philosophical issues, of static and dynamic realities, of objective and subjective perspectives, of inductive and deductive approaches or of integral and particular visions. It is not the elusive mix of numerical and text data or of simultaneous considerations of antagonistic approaches of causality and non-causality. Integration of data does not occur in the analysis process, but in the union of the results of each study within a cohesive and coherent product where the confirmation or revision of the existing theory takes place. This can be achieved through adhesion to the rules and assumptions of each method in selecting the sample, purpose, method, and the contribution of the results within the research plan as a whole.

## References

[1] Boudon R. (1989). Os métodos em sociología.

[2] Morse SM. (1991). Approaches the Qualitative-Quantitative Metodological Triangulation, Metodology Corner. Rev. Nur. Res.

[3] Cowman S. (1993). Triangulation: a means of reconciliation in nursing research. J. Adv. Nurs..

[4] Denzin NK., Denzin NK. (2017). The Research Act:. A theoretical Introduction to Sociological Methods.

[5] Kimchi J, Polivka B, Stevenson JS. (1991). Triangulation: Operational Definitions. Nurs. Res..

[6] Tobin G., Begley C. (2004). Methodological rigour within a qualitative framework. J. Adv. Nurs..

[7] Flick U. (2014). La gestión de la calidad en la investigación cualitativa.

[8] Patton M. (1999). Enhancing the Quality and Credibility of Qualitative Analysis. Health Serv. Res..

[9] Flick U. (2017). Mantras and Myths: The Disenchantment of Mixed-Methods Research and Revisiting Triangulation as a Perspective. Qual. Inq..

[10] Creswell J. (2014). Research Design QualQuan and Mixed Methods.

[11] Janesick V., Denman CA, Haro JA (2000). Por los rincones. Antología de métodos cualitativos en la investigación social.

[12] Molina G. (2020). Integración de métodos de investigación. Estrategias metodológicas y experiencias en Salud Pública.

[13] Timans R, Wouters P, Heilbron J. (2019). Mixed methods research: what it is and what it could be. Theory Soc..

[14] Mitchell ES. (1986). Multiple Triangulation: a methodology for nursing science. Adv. Nurs. Sci..

